# Detection of *EGFR* Mutations Using Bronchial Washing-Derived Extracellular Vesicles in Patients with Non-Small-Cell Lung Carcinoma

**DOI:** 10.3390/cancers12102822

**Published:** 2020-09-30

**Authors:** Juhee Park, Chaeeun Lee, Jung Seop Eom, Mi-Hyun Kim, Yoon-Kyoung Cho

**Affiliations:** 1 Center for Soft and Living Matter, Institute for Basic Science (IBS), Ulsan 44919, Korea; jugene@nate.com (J.P.); chaeeun@unist.ac.kr (C.L.); 2 Department of Biomedical Engineering, Ulsan National Institute of Science and Technology (UNIST), Ulsan 44919, Korea; 3 Department of Internal Medicine, Pusan National University School of Medicine and Biomedical Research Institute, Pusan National University Hospital, 179, Gudeok-ro, Seo-Gu, Busan 49241, Korea; ejspulm@gmail.com

**Keywords:** lung cancer, liquid biopsy, bronchial washing (BW), extracellular vesicles, EGFR, T790M

## Abstract

**Simple Summary:**

Considering the spatiotemporal heterogeneity, more frequent monitoring of the disease progress using less-invasive liquid biopsy technologies is highly desired. Here, we demonstrate that epidermal growth factor receptor (*EGFR*) mutations could be readily detected from minimally invasive bronchial washing (BW)-derived EVs with good accuracy. The acquisition of T790M resistance mutation was detected earlier in BW-derived EVs than in plasma or tissue samples. The longitudinal analysis of BW-derived EVs showed excellent correlation with the disease progression measured by CT images. We demonstrate the clinical potential of BW-derived EVs as a liquid-biopsy sample for prognosis and precision medicine in patients with lung cancer.

**Abstract:**

The detection of epidermal growth factor receptor (*EGFR*) mutation, based on tissue biopsy samples, provides a valuable guideline for the prognosis and precision medicine in patients with lung cancer. In this study, we aimed to examine minimally invasive bronchial washing (BW)-derived extracellular vesicles (EVs) for *EGFR* mutation analysis in patients with lung cancer. A lab-on-a-disc equipped with a filter with 20-nm pore diameter, Exo-Disc, was used to enrich EVs in BW samples. The overall detection sensitivity of *EGFR* mutations in 55 BW-derived samples was 89.7% and 31.0% for EV-derived DNA (EV-DNA) and EV-excluded cell free-DNA (EV-X-cfDNA), respectively, with 100% specificity. The detection rate of T790M in 13 matched samples was 61.5%, 10.0%, and 30.8% from BW-derived EV-DNA, plasma-derived cfDNA, and tissue samples, respectively. The acquisition of T790M resistance mutation was detected earlier in BW-derived EVs than plasma or tissue samples. The longitudinal analysis of BW-derived EVs showed excellent correlation with the disease progression measured by CT images. The *EGFR* mutations can be readily detected in BW-derived EVs, which demonstrates their clinical potential as a liquid-biopsy sample that may aid precise management, including assessment of the treatment response and drug resistance in patients with lung cancer.

## 1. Introduction

Lung cancer is one of the leading causes of cancer-related deaths worldwide, with the highest cancer-related death rate [1,2,3]. Recent advances in precision medicine have had profound implications on the diagnostics and treatment monitoring in patients with lung cancer [4,5,6]. The somatic mutations found in cancer tissues strongly influence the patients′ survival rate, and the best treatment options for individual patients can be determined by clinicians according to the molecular profiling of the cancer [7,8,9].

Genetic mutation of epidermal growth factor receptor (*EGFR*) gene is one of the critical therapeutic markers for lung cancer [10]. It is related to tyrosine kinase inhibitor (TKI)-associated drugs [11,12], which inhibit the abnormal growth of tumor by reducing the binding of tyrosine kinase with EGFR in the cancer cells. The T790M mutation of the EGFR tyrosine kinase domain constitutes a large portion of resistance to these drugs. When the T790M mutation of the *EGFR* gene is timely detected, the resistance to the TKI drug can be overcome by selecting alterative options to improve the outcomes of patients with lung cancer [13,14,15].

Even better, liquid biopsy, which is now frequently used in non-invasive prenatal diagnostic test area, is expanding toward the diagnosis and treatment in cancer. Liquid biopsy is relatively less invasive and complementary to the tissue biopsy, which is invasive and sometimes not a feasible option in several patients [16,17,18]. Various circulating biomarkers such as circulating tumor cells (CTCs), cell-free DNA (cfDNA), and extracellular vesicles (EVs) have been used as source materials for the characterization of tumors. EVs are small vesicles (30–1000 nm) surrounded by a lipid bilayer, secreted or protruded by the cells [19,20]. It has been reported that EVs released by cancer tissues contain tumor-associated genetic materials such as mRNA, DNA, and proteins [21,22,23]. While CTCs and cfDNA have been mainly used for liquid biopsy applications [24,25,26], EVs can be potentially superior [27] because their contents such as genetic materials could remain protected by its lipid bilayer [28,29]. EVs have been associated with the intercellular communication and cancer metastasis [30,31]. Therefore, we hypothesize that monitoring tumor-derived EVs can be an efficient way to obtain dynamic information on cancer progression in patients undergoing therapy.

The current gold standard for detecting *EGFR* mutation in lung cancer patients in the clinic is cell cytology using tissue biopsy samples [25,32]. Recently reported studies used EVs and cfDNA for lung cancer detection via plasma obtained from patients [16,33]. Additionally, *EGFR* mutations were detected from DNA obtained from EVs in bronchoalveolar lavage fluid (BALF) of non-small-cell lung cancer (NSCLC) patients [34]. However, the current drawbacks of using BALF or plasma are that BALF shows higher detection rate but is relatively invasive and thus not a routine procedure of bronchoscopy [35], while plasma is less invasive but shows lower detection rate compared to BALF [34].

Flexible bronchoscopy (FB) is an essential step in the diagnosis of lung cancer. Many diagnostic procedures using FB, including washing, brushing, bronchoalveolar lavage, or biopsy, have been used in various combinations to improve the diagnostic yield of FB in patients with suspected lung cancer. BALF is obtained by advancing the bronchoscope into an airway, instilling about 100–150 mL of sterile saline, and then quickly aspirating the instilled saline into a specimen trap. Bronchial washing (BW) samples are similar to BALF, but different in that they need smaller volume of sterile saline and do not require that the bronchoscope be wedged [36]. BW is widely known as a safe method [37,38,39].

There are many methods for isolating EVs such as ultracentrifugation, immunoaffinity-based isolation, size exclusion chromatography, and ultrafiltration [40]. In this study, we focused on the BW samples for less invasive sampling and enriched EVs for more sensitive molecular detection of *EGFR* mutations. Using a size-based EVs filtration method, we analyzed EV-derived DNA (EV-DNA) and EV-excluded cell free-DNA (EV-X-cfDNA) from BW for the mutation detection in lung cancer. *EGFR* mutations, including L858R, 19del, and T790M, were examined in both EV-DNA and EV-X-cfDNA from BW samples. Furthermore, the detection rate of T790M in BW-derived EVs was compared with that in tissue, and blood plasma-based samples.

## 2. Results

### 2.1. Patients

From July 2018 to September 2019, 52 lung cancer patients were enrolled in this study. Among them, 26 patients were mutant-type and 26 patients were wild-type according to the clinical tissue-based detection. The median age of patients was 66 (ranging from 39 to 89) years for mutant types and 68 (ranging from 50 to 81) years for wild types. The clinical information of all patients is listed in Table 1. The 26 mutant-type cases showed 38.5% of L858R and 61.5% of 19del mutation based on histological and pathological data. Four patients with L858R or 19del mutation showed acquired resistance to EGFR-TKI according to tissue re-biopsy, during the TKI treatment. BW samples were collected from all patients and used for *EGFR* mutation detection by separately isolating EV-DNA and EV-X-cfDNA.

### 2.2. Detection of EGFR Mutation in Bronchial Washing-Derived Samples

**Figure 1:**
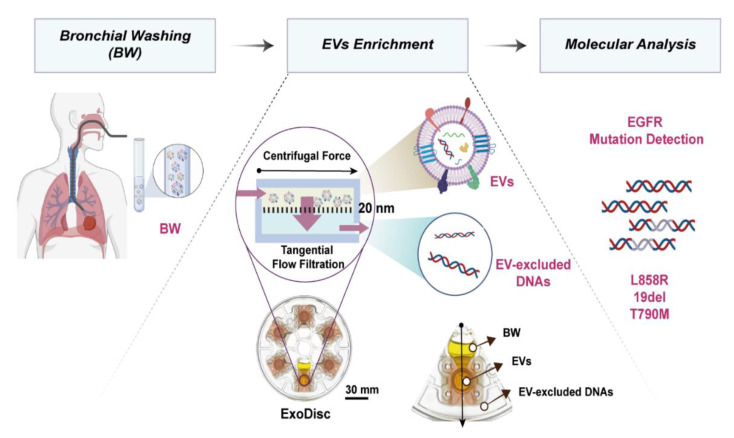
Schematic illustration of experimental process. Bronchial washing (BW) samples are injected into an Exo-Disc. Upon spinning the disc, the liquid flow through the filter that is in tangential direction with the centrifugal force minimizing the clogging issue. The extracellular vesicles (EVs) are enriched on top of the filter by size-based isolation and the flow-through liquid is used to analyze the EV-excluded cell free DNA. The EGFR mutation is confirmed by Polymerase Chain Reaction.

**Figure 2:**
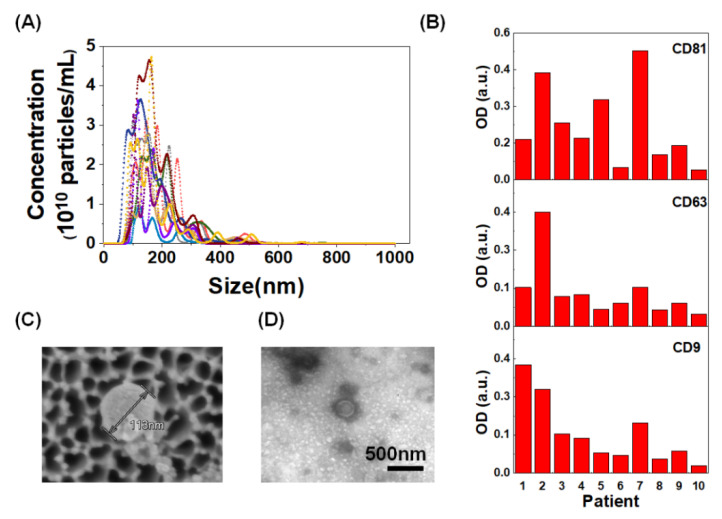
Characterization of extracellular vesicles (EVs): (**A**) size distribution and concentration of EVs measured by Nanoparticle Tracking Analysis; (**B**) EVs were confirmed by ELISA using CD9, CD63, and CD81 markers; and (**C**) scanning electron microscope and (**D**) transmission electron microscope images of bronchial washing-derived-EVs.

We enriched EV-DNA in BW samples (<4 mL) using Exo-Disc [41]. EV-X-cfDNA were collected in the flow-through chamber of the device after the filtration of EVs. Then, *EGFR* mutation detection was confirmed by real-time PCR and ddPCR by targeting L858R, 19del, and T790M using EV-DNA and EV-X-cfDNA (Figure 1). To confirm that we could detect the EVs from the BW, 10 samples were analyzed. After the isolation of EVs, their characteristics were confirmed using size, shape, and indirect enzyme-linked immunosorbent assays (ELISA) to measure EV markers such as tetraspanins CD9, CD63, and CD81 (Figure 2). The average size of EVs was 205.1 ± 7.3 nm, and the particle concentration was 1.7 × 10^10^–2.2 × 10^11^ particles/mL.

In total, 55 BW samples from 52 patients having 29 mutant and 26 wild type results from tissue samples were subject of the study to prepare EV-DNA and EV-X-cfDNA samples. As shown in Table 2, of the 55 samples, the L858R and 19del *EGFR* mutations were identified by real time PCR in 26 EV-DNA samples and 9 EV-X-cfDNA samples, respectively. The wild-type *EGFR* was detected in 26 EV-DNA and 26 EV-X-cfDNA samples. Therefore, the *EGFR* mutation detection sensitivity was 89.7% and 31% from EV-DNA and EV-X-cfDNA, respectively, with 100% specificity in both groups. 

### 2.3. EGFR T790M Mutation Detection in Bronchial Washing-Derived, Plasma, and Tissue Samples

**Figure 3:**
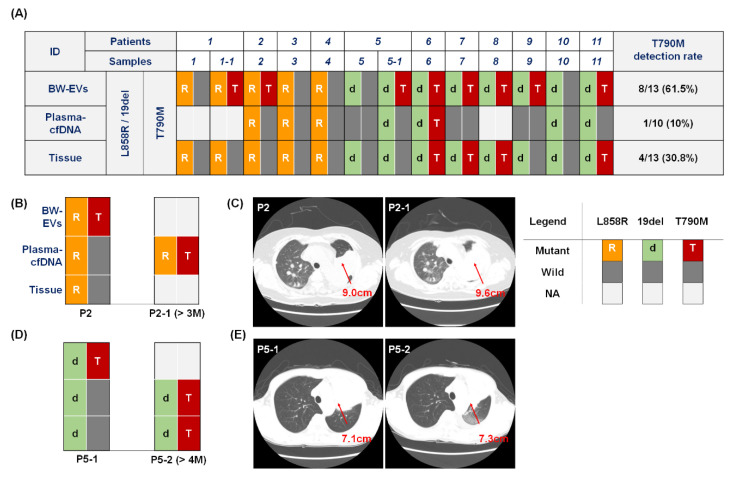
Detection of original mutations (L858R, 19del) and acquired T790M by ddPCR in BW-derived EV-DNA with plasma-derived cfDNA and tissue samples. (**A**) In 11 patients with re-biopsy samples, T790M was detected in 61.5%, 10%, and 30.8% of EV-DNA in BW, cfDNA of plasma, and tissues, respectively. Patients 1 (P1) and 5 (P5) were included in the longitudinal study and additional samples were collected after four and eight months for P1 (P1-1) and P5 (P5-1), respectively. (**B**–**E**) In samples of P2 (**B**,**C**) and P5-1 (**D**,**E**), T790M was detected in BW-derived EV-DNA samples but not in tissue samples. After three or four months, the acquired T790M mutation was detected from plasma cfDNA (P2-1) and both from plasma cfDNA and tissue re-biopsy samples (P5-2), respectively. CT scan images showed that the tumor size slightly increased (**C**) from 9.0 (P2) to 9.6 cm (P2-1) and (**E**) from 7.1 (P5-1) to 7.3 cm (P5-2) during the three and four months for P2 and P5-1, respectively.

We tested *EGFR* T790M mutation in BW-derived EV-DNA by ddPCR and compared that with the corresponding tissue samples and cfDNA derived from plasma in 11 patients. As shown in Figure 3A, *EGFR* T790M mutations were identified in eight BW EV-DNA samples, while only one case from blood plasma derived cfDNA and four tissue samples showed T790M mutations. Therefore, the T790M detection rate was 61.5%, 10.0%, and 30.8% in BW-derived EV-DNA, cfDNA from plasma, and tissues, respectively. Notably, eight cases of acquired T790M mutation were detected among 11 patients via the BW-derived EV-DNA samples, including those four patients whose tissue sample showed the T790M mutation (P6, P7, P8, and P11). In Patient 6, the acquired T790M was detected via all samples, i.e., BW-derived EV, blood plasma cfDNA, and tissue samples. Furthermore, L858R and 19del *EGFR* mutations were identified by ddPCR in 11 BW-derived EVs and the corresponding tissue samples, which resulted in 100% sensitivity. Conversely, in the case of plasma-derived cfDNA, three of 10 available samples were false negative (P5, P7, and P9) in the detection of L858R and 19del *EGFR* mutations, which resulted in 70% sensitivity. When we compared the number of copies of the *EGFR* mutations (L858R and 19del) found in 13 BW-derived EV-DNA samples and the corresponding tumor size measured from CT scan images, there was weak positive correlation (Figure S1).

We repeated the tests using the available samples from the patients who showed the acquired T790M mutation in only BW EV-DNA samples but not in the tissue samples (P2 and P5-1). In the cases of P1-1 and P9, the follow-up samples were not available because of the death or transfer to other hospital. In Patient 2, the T790M mutation could be detected in BW EV-DNA sample initially but not in both the tissue and blood plasma derived cfDNA samples (P2). After three months (P2-1), however, the T790M mutation could be detected in the plasma cfDNA samples, confirming the earlier detection of T790M from the BW EV-DNA samples (Figure 3B). CT scan images showed that the tumor size slightly increased from 9.0 (P2) to 9.6 cm (P2-1) during the three months (Figure 3C). The corresponding tissue sample after three months could not be achieved because of the patient′s condition.

Similarly, the 19del mutation was identified in both BW EV-DNA and tissue samples but not in plasma-derived cfDNA in Patient 5 (P5). Four months later, the 19del *EGFR* mutations could be detected from all of the samples, while the T790M mutation was detected only in BW EV-DNA samples (P5-1) but not in plasma cfDNA and tissue samples. However, four months later (P5-2), the T790M mutations were detected from both plasma-derived cfDNA and the corresponding tissues samples, which confirmed the earlier detection of T790M mutation in BW EV-DNA samples than plasma cfDNA or tissue samples (Figure 3D). CT scan images showed that the tumor size slightly increased from 7.1 (P5-1) to 7.3 cm (P5-2) during the four months (Figure 3E).

### 2.4. Monitoring of EGFR Mutation during Targeted Therapy

**Figure 4:**
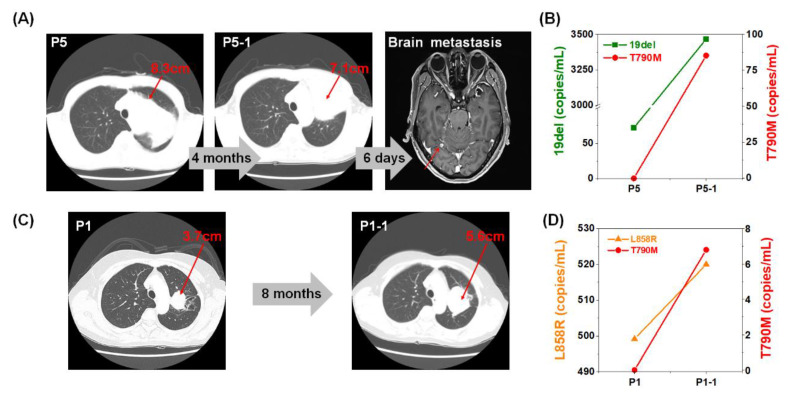
Monitoring of lung cancer patients via CT scan image and molecular analysis of EGFR mutations by ddPCR of BW-derived EV-DNA. (**A**) The size of the tumor decreased from 8.3 (P5) to 7.1 cm (P5-1) after about four months. However, a new metastasized lesion was found in the brain. (**B**) 19del EGFR mutation was increased and T790M EGFR mutation was emerged. (**C**) The tumor size increased from 3.7 to 5.6 cm after eight months of TKI therapy. (**D**) The L858R EGFR mutation was also increased, and T790M EGFR mutation emerged from ddPCR results of BW-derived EV-based liquid biopsy.

In the longitudinal monitoring of BW-derived EV-DNA samples performed in a blind manner, we observed meaningful prognostic value of BW-derived EV-DNA-based genotyping. The number of copies of the *EGFR* mutations found in BW-derived EV-DNA from a 61-year-old male (P 5), who had stage IIIB, SCC with 19del mutation, increased from 72 to 3466.7 copies/mL for the 19del mutation, and from 0 to 85.3 copies/mL for the T790M mutation, respectively (Figure 4A,B). While the primary tumor size decreased from 8.3 to 7.1 cm during the four-month period of monitoring, additional CT image taken afterwards could confirm the presence of a new metastasis in the brain. In this example, the increase of the EGFR T790M mutation in EV-DNA from the BW-derived sample could be detected even though the primary tumor size in the CT image was decreased, which was much earlier than the CT image could identify the metastasis to the brain. In another case (P 1) of longitudinal monitoring of BW-derived samples, ddPCR data showed the increase of EGFR L858R mutations, and the emergence of T790M mutations. The L858R mutation increased from 499.2 to 520 copies/mL, and the T790M mutation emerged from 0 to 6.8 copies/mL, respectively (Figure 4C,D). As we checked the corresponding patients’ information, we learned that the BW samples were from a 52-year-old female with stage IVB of adenocarcinoma with L858R mutation. According to the CT images taken about eight months after the diagnosis, the tumor size increased from 3.7 to 5.6 cm. In both patients, the treatment was changed from afatinib to osimertinib after the resistance gene T790M was detected. 

## 3. Discussion

Sensitive and frequent genotyping of the *EGFR* mutation can benefit patients with NSCLC by providing timely guidelines on the appropriate EGFR-targeted therapies. To the best of our knowledge, this was the first study to report that *EGFR* mutations could be readily detected from BW-derived EV-DNA samples with good accuracy (94.5%). The *EGFR* mutation detection sensitivity, 26/29 (89.7%), that we achieved using BW-derived EV-DNA samples was slightly lower than the previous literature report using more invasive BALF-derived EV-DNA samples, 14/14 (100%) [34]. However, it was higher than the plasma-derived EV-DNA samples from patients with early stage 38/148 (26%), or late-stage (~80%) NSCLC [42], demonstrating that the proximal fluid may more accurately represent the tumor status. Although the BW-derived samples have been previously regarded as less sensitive compared to BALF, our results show that BW-derived EVs can be an indispensable source for the liquid biopsy considering the tumor contiguity, and because it is less invasive and can be performed with lower risk for both the patient and clinician. 

This study had several potential limitations. First, this was a single-center study with available samples from only 52 patients. Second, even though we tried to compare the detection rate of the *EGFR* mutation from BW-derived EVs, plasma-derived cfDNA, and tissue samples, we could only have 11 cases of perfectly matched samples among the 26 patients because the clinical samples were not always available depending upon the health conditions of the patients. Third, we only tested the selected mutations including 19del, L858R, and T790M mutations in *EGFR*. Fourth, it was a prospective study, and we could not track the patients’ prognosis and overall survival time. Therefore, we could not investigate the correlation between the mutation detected from BW-derived EVs and the response to the therapy and their survival outcomes. Finally, we could collect liquid biopsy samples only when the patients visited the hospital for their regular check-up and CT scan imaging, even though, under ideal circumstances, the liquid biopsy could be performed more frequently.

## 4. Materials and Methods 

### 4.1. Storage and Handling of Clinical Samples

In this prospective study, BW fluids were obtained through bronchoscopy with written informed consent from all participants. BW samples were collected from patients diagnosed with non-small cell lung cancer by a pathologist; samples from 26 patients who were *EGFR*-negative and 26 patients who were *EGFR*-positive were collected. The study protocol was reviewed and approved by the Institutional Review Board (IRB) of Pusan National University Hospital (IRB 1805-031-067). Samples were centrifuged at 300× *g* for 10 min, 2000× *g* for 10 min at 4 °C to remove cell and debris, and stored at −80 °C before use.

### 4.2. Bronchoscopy and Process of Bronchial Washing 

The bronchoscopies were done trans-orally or trans-nasally using a flexible fiber-optic bronchoscope (Olympus, Tokyo, Japan) after sedation with midazolam and fentanyl and were examined by board-certified respiratory therapist. Oropharyngeal and laryngeal anesthesia was induced by administrating 2 mL of nebulized 4% lidocaine before the bronchoscopy, followed by spraying 1 mL of 2% topical lidocaine into the oral and nasal cavities. The bronchoscope was inserted through the vocal cords, followed by instillation of 2% lidocaine solution into the trachea and bilateral main bronchi through the working channel. After the initial exploration, bronchial washing samples were collected by instilling 10 mL of sterile normal saline followed by immediate suction thereafter.

### 4.3. Isolation of Extracellular Vesicles

EVs were isolated using a previously described lab-on-a-disc platform, Exo-Disc [41,43,44]. Each disc is composed of 6 sample chambers each of which is connected to an AAO membrane with 20 nm pore size for highly efficient tangential flow filtration. BW sample was injected into the sample chamber after a pre-filtration step using a 0.8 μm syringe filter. The disc was rotated at a speed of 500× *g* to isolate EVs. The EV excluded BW sample was filtered out in flow-through chamber and collected separately (Figure 1). Before washing, to remove free-floating DNA that remained outside of EVs, EVs solution was incubated with 50 U DNase (Qiagen, Hilden, Germany) in phosphate buffered saline (PBS) for 20 min at room temp (RT), and washed with PBS at 500× *g*, the isolated EVs were eluted in 100 μL of prefiltered PBS and used for downstream analysis.

### 4.4. Nanoparticle Tracking Analysis

Nanoparticle tracking analysis (NTA) was done using Nanosight NS 500 system (Malvern Instruments, Malvern, UK) to measure the size distribution and concentration of EVs. EV samples were gently resuspended and diluted with pre-filtered PBS according to the manufacturer’s recommended concentration range (25–100 particles/frame). To ensure consistency, identical settings were used for all measurements and the mean values were calculated after the quintuplet replicates.

### 4.5. Confirmatory Indirect ELISA for Markers of Extracellular Vesicles

ELISA was performed using a 96-well plate (Corning Inc., Steuben County, NY, USA). EVs in PBS (50 µL) were used to coat a well by incubating it overnight at 4 °C; 1% BSA (bovine serum albumin) in PBS was treated at RT for 1 h to prevent nonspecific binding. After washing with 0.1% BSA-PBS buffer, the plate was incubated with primary antibodies CD9 (Cat No. 555370, BD Pharminogen), CD63 (Cat No. 556019), and CD81 (Cat No. 555675) in PBS at RT for 1 h. After washing three times with the washing buffer, the plate underwent 1 h incubation with HRP-conjugated secondary antibodies at RT. The plate was washed three times with washing buffer and incubated for 15 min at RT after adding TMB solution. The stop solution was added, and the absorbance of each solution was measured using the Infinite M200 plate reader (Tecan, Männedorf, Switzerland) at 450 nm.

### 4.6. DNA Isolation from Bronchial Wash Sample

For EV-DNA and EV-X-cfDNA isolation, EVs were isolated from 2–4 mL of bronchial wash sample by Exo-Disc. The EV-X-cfDNA samples were collected from the flow-through chamber. Allprep DNA/RNA kit (Qiagen, Hilden, Germany) and QIAamp circulating nucleic acid kit were used to extract EV-DNA and EV-X-cfDNA, respectively. 

### 4.7. Cell-free DNA Isolation from Plasma

Around 5 mL of blood was collected in an EDTA-treated tube. cfDNA was extracted from plasma using Cobas^®^ cfDNA Sample Preparation Kit (Roche Molecular Systems, Pleasanton, CA, USA). Extracted DNA was examined to find mutations using the protocol for cfDNA, with Cobas^®^ EGFR Mutation assay.

### 4.8. Real-Time Polymerase Chain Reaction

For the mutation analysis, 19del, L858R, and T790M were confirmed by PANAMutyper EGFR mutation detection kit (Panagene, Daejeon, Korea). Five microliters of DNA template, 19 μL mastermix, and 1 μL Taq DNA polymerase were used. Polymerase chain reaction (PCR) amplification was performed using the QuantStudio6 (Thermo Fisher Scientific, Waltham, MA, USA) per the following protocol: 50 °C for 2 min and 95 °C for 15 min; 15 cycles 95 °C for 30 s, 70 °C for 20 s, and 63 °C for 1 min; 35 cycles of 95 °C for 10 s, 53 °C for 20 s, and 73 °C for 20 s; and then, for melting curve analysis, followed by 95 °C for 15 min, 35 °C for 5 min, and 35 to 75 °C (increment 0.5 °C) for 3 s.

### 4.9. Droplet Digital PCR 

L858R, 19del, and T790M mutations of the *EGFR* gene were detected by droplet digital PCR (ddPCR). The samples were prepared in 20 μL of reaction volume, including 10 μL of ddPCR Super Master Mix, 5 μL of DNA template, and 1 μL each of the primer/probe. The primer/probes were purchased from Bio-Rad (BR186dHsaCP 2000019, 2000020, 2000021, 2000022, 2000039, and 2000040). Droplets were generated using a QX200 droplet generator (Bio-Rad, Hercules, CA, USA) using 70 μL of oil. Master cycler pro S (Eppendorf, Hamburg, Germany) was used for PCR amplification employing the following protocol: 95 °C for 10 min; 40 cycles of 94 °C for 30 s, 58 °C for 1 min, and 98 °C for 10 min; and then held at 4 °C. The fluorescence signal was measured with a QX200 droplet reader (Bio-Rad) and analyzed by QuantaSoft software (Bio-Rad).

### 4.10. Scanning Electron Microscope Imaging 

Each membrane with the enriched EVs was fixed using 4% paraformaldehyde in PBS at RT for 20 min. Subsequently, the surface of each membrane was washed once with PBS and went through serial dehydration steps with 50%, 70%, 90%, and 100% of ethanol for 10 min each. Finally, 100% ethanol treatment was repeated once again and dried at RT. The EVs fixed on the membrane were attached to the sample holder with carbon paste, and the surface was coated with Pt by sputter. The scanning electron microscope (SEM) images of EVs were acquired with SU-8220 cold FE-SEM instrument (Hitachi high technologies, Tokyo, Japan).

### 4.11. Bio-Transmission Electron Microscope Imaging

EVs were fixed with 4% formaldehyde after isolation and then fixed EVs were dropped on the pre-cleaned parafilm. Poly-L-Lysine (PLL) coated formvar/carbon film grids were flipped on the drop to absorb the sample. After flipping the grids upside down, they were dried at RT for 30 min. The dried samples were stained with 10 μL of uranyless EM Stain for 1 min. The remaining solutions were absorbed by paper and then dried. The image was taken by JEM-1400 Bio-Transmission Electron Microscope (JEOL, Tokyo, Japan).

## 5. Conclusions

We demonstrated that BW-derived EVs could be used for accurate and frequent genotyping of *EGFR* mutation in patients with NSCLC, and for early detection of actionable mutations for appropriate therapy selection and monitoring of disease progression. Emerging T790M mutation could be detected from BW-derived EV-DNA samples with superior detection rate to that in blood plasma-derived cfDNA or tissue biopsy samples. Considering the importance of the early detection of T790M, which is the marker related with TKI-related drug resistance, we carefully raise the necessity that a larger study with EV-DNA from BW-derived samples may prove to be a more effective way of mutation detection to predict and monitor the patients’ response to treatment and suggest appropriate treatment change in the therapeutic regimen. Overall, we anticipate that the liquid biopsy using BW-derived EVs, enriched by using Exo-Disc, may promote frequent monitoring of tumor progression and contribute to the broader application of personalized medicine.

## Figures and Tables

**Table 1:** Patient’s clinical and pathological characteristics.

Characteristic (*n* = 52)	Mutant Type (%) *n* = 26	Wild Type (%) *n* = 26
Median age (range)	66 (39–89)	68 (50–81)
Sex		
Male	10 (38.5)	24 (92.3)
Female	16 (61.5)	2 (7.7)
Histology		
Adenocarcinoma	23 (88.5)	10 (38.5)
Squamous cell carcinoma	2 (7.7)	16 (61.5)
Non-small-cell lung carcinoma, NOS ^1^	1 (3.8)	0 (0)
EGFR mutation type		
L858R	10 (38.5)	0 (0)
19del	16 (61.5)	0 (0)
Stage, *n* (%)		
I	0 (0)	3 (11.5)
II	2 (7.7)	4 (15.4)
III	2 (7.7)	6 (23.1)
IV	22 (84.6)	11 (42.3)
Unknown	0 (0)	2 (7.7)

^1^ NOS, not otherwise specified.

**Table 2:** Detection rate of EGFR mutation by real time PCR using bronchial washing sample; EV-DNA and EV-excluded-cell free DNA (EV-X-cfDNA) from bronchial washing sample.

EGFR Mutation	Tissue	BW (*n* = 55)	
		EV-DNA	EV-X-cfDNA
Mutant	29	26	9
Wild	26	26	26
Sensitivity		89.7%	31.0%
Specificity		100%	100%

## Supplementary Materials

The following are available online at https://www.mdpi.com/2072-6694/12/10/2822/s1, Figure S1: The number of copies of the *EGFR* mutations (L858R and 19del) found in 13 BW-derived EV-DNA samples and the corresponding tumor size measured from CT scan images
