# Supplementary material for: Detection of EGFR Mutations Using Bronchial Washing-Derived Extracellular Vesicles in Patients with Non-Small-Cell Lung Carcinoma
Source: Cancers (Basel). 2020 Sep 30;12(10):2822. doi: 10.3390/cancers12102822 (PMC7599768; doi:10.3390/cancers12102822)
Supplement: Supplementary file 1 [file cancers-12-02822-s001.pdf]

Detection of *EGFR* mutations using bronchial washing-derived extracellular vesicles in patients with non-small-cell lung carcinoma

Juhee Park, Chaeeun Lee, Jung Seop Eom, Mi-Hyun Kim and Yoon-Kyoung Cho

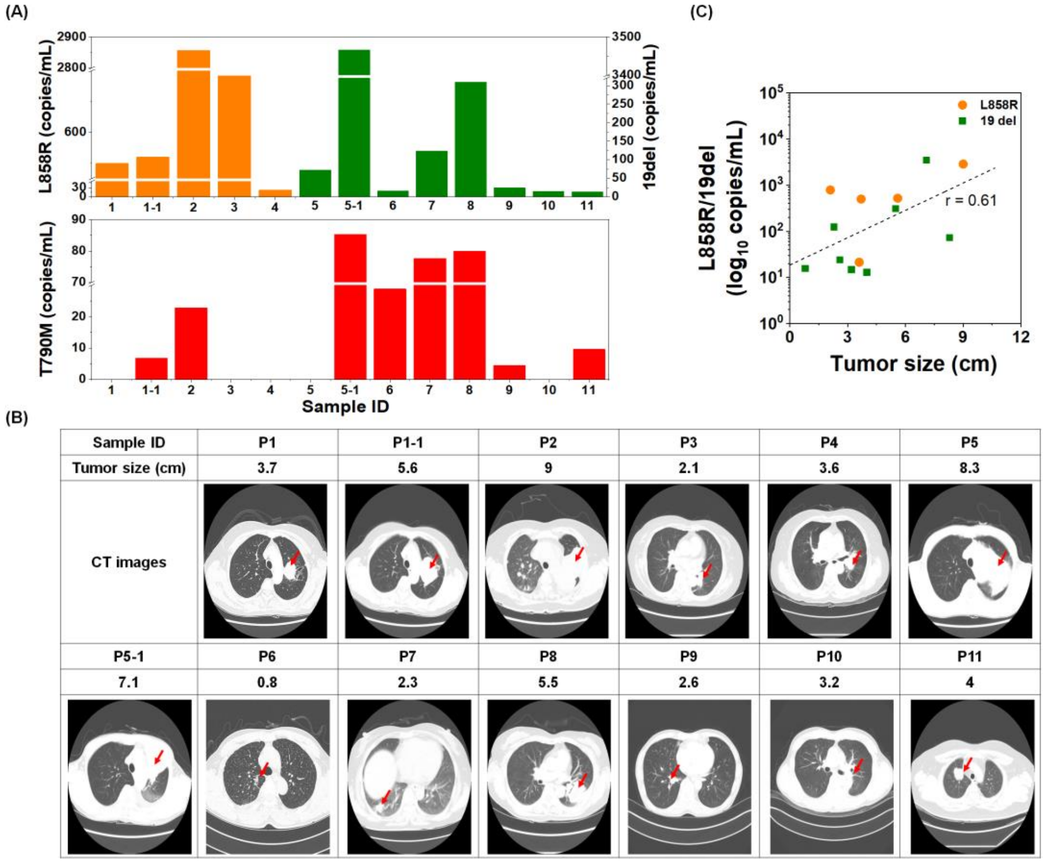

Figure S1: (**A**) The number of copies of the *EGFR* mutations (L858R, 19del, and T790M) found in 13 BW-derived EV-DNA samples and (**B**) the corresponding tumor size measured from CT scan images. (**C**) There was weak positive correlation between the tumor size and the number of copies of *EGFR* mutations of L858R and 19del (Pearson's r Correlation (r) = 0.61, p = 0.03).

© 2020 by the authors. Licensee MDPI, Basel, Switzerland. This article is an open access article distributed under the terms and conditions of the Creative Commons Attribution (CC BY) license (http://creativecommons.org/licenses/by/4.0/).
